# Broadband Unidirectional Forward Scattering with High Refractive Index Nanostructures: Application in Solar Cells

**DOI:** 10.3390/molecules26154421

**Published:** 2021-07-22

**Authors:** Ángela Barreda, Pablo Albella, Fernando Moreno, Francisco González

**Affiliations:** 1 Abbe Center of Photonics, Institute of Applied Physics, Friedrich Schiller University Jena, Albert-Einstein-Str. 15, 07745 Jena, Germany; 2 Group of Optics, Department of Applied Physics, University of Cantabria, Cantabria, 39005 Santander, Spain; pablo.albella@unican.es (P.A.); morenof@unican.es (F.M.); gonzaleff@unican.es (F.G.)

**Keywords:** high refractive index dielectric nanoparticles, scattering directionality conditions, solar cells

## Abstract

High refractive index dielectric (HRID) nanoparticles are a clear alternative to metals in nanophotonic applications due to their low losses and directional scattering properties. It has been demonstrated that HRID dimers are more efficient scattering units than single nanoparticles in redirecting the incident radiation towards the forward direction. This effect was recently reported and is known as the “near zero-backward” scattering condition, attained when nanoparticles forming dimers strongly interact with each other. Here, we analyzed the electromagnetic response of HRID isolated nanoparticles and aggregates when deposited on monolayer and graded-index multilayer dielectric substrates. In particular, we studied the fraction of radiation that is scattered towards a substrate with known optical properties when the nanoparticles are located on its surface. We demonstrated that HRID dimers can increase the radiation emitted towards the substrate compared to that of isolated nanoparticles. However, this effect was only present for low values of the substrate refractive index. With the aim of observing the same effect for silicon substrates, we show that it is necessary to use a multilayer antireflection coating. We conclude that dimers of HRID nanoparticles on a graded-index multilayer substrate can increase the radiation scattered into a silicon photovoltaic wafer. The results in this work can be applied to the design of novel solar cells.

## 1. Introduction

High refractive index dielectric (HRID) nanoparticles (NPs) have been suggested as an alternative to metallic ones, due to their low losses in the VIS and NIR spectral regions [1,2,3,4,5,6,7,8,9,10,11] and their interesting directionality properties. The latter are a consequence of the coherent effects between the electric and magnetic resonances observed in their spectral response, in spite of their being non-magnetic materials [2]. In particular, under the so-called Kerker’s conditions [12,13,14,15,16,17,18,19], the scattered radiation from a single HRID spherical nanoparticle can be scattered either towards the backward or forward regions at the near zero-forward or zero-backward conditions, respectively. In previous works [19,20,21], we evidenced, theoretically and experimentally, that subwavelength symmetric and antisymmetric dimers of HRID particles can be efficient scattering units for redirecting the incident radiation into the forward direction. We also showed [20,21] that, for strong interaction effects between the particles forming the dimer (small gaps), it is possible to find (within the spectral range where the dipolar approximation holds) two spectral regions where the incident radiation can be scattered forward. Those regions correspond to the zero-backward condition and to a new scattering directionality condition (SDC) denoted as the “near zero-backward” condition, which is a 180° rotated version of the traditional near zero-forward condition [20,21]. For isolated HRID nanoparticles, only the zero-backward condition can be observed. This reveals the dimer as a more efficient configuration for boosting unidirectional forward scattering in a broadband spectral range [20].

One of the requirements to make solar cells economically competitive with respect to other energy sources is the reduction in thickness of the photosensitive silicon layer. However, in thin films, solar energy is poorly absorbed. The use of metallic nanoparticles was proposed as an attempt to address this issue. These NPs can increase the absorption of the incident electromagnetic radiation by exploiting both near- and far-field properties [22,23]. On the one hand, light scattering is increased by the NPs, spreading the electromagnetic radiation across a wider angular range. As a consequence, the optical path length of the radiation in the substrate is increased. On the other hand, the enhancement of the electromagnetic energy in the nanoparticle surroundings boost the absorption of the incident radiation in the silicon photosensitive layer. However, the inherent ohmic losses of metallic NPs limit their application. The low losses of HRID nanoparticles, together with their directional capabilities, make them interesting scattering units to improve the performance of solar cells [10,11,24,25]. An issue with isolated spherical NPs to enhance the performance of solar cells is that the efficiency of the zero-backward condition is low. This is due to the interference between the dipolar electric and magnetic resonances—responsible for directionality conditions—being attained at the tails of the resonances [14]. In order to boost the scattering efficiency of the zero-backward condition, different geometries of isolated HRID NPs have been proposed, namely cylinders [26] and spheroids [27]. In [25], we analyzed distinct shapes of HRID NPs (spheres, cylinders, ellipsoids oblate and prolate, and parallelepipeds) to identify the configuration that provides the strongest scattering in the forward direction with null backward scattering. Aggregates of HRID NPs have been proposed with the same aim, notably asymmetric dimers [19]. Despite the proposed configurations improving the efficiency of the zero-backward condition, unidirectional forward scattering is only attained in a small spectral region. For solar cell applications, which require a broadband response, it is necessary to achieve the scattering directionality conditions at different ranges of wavelengths. Here, we analyzed the possibility of using aggregates of HRID NPs with strong interaction effects between their components to attain two spectral regions where the incident radiation is scattered towards the photovoltaic substrate. One of them corresponds to the wavelengths where the zero-backward condition is observed (also achieved for isolated particles), and the other is associated with the “near zero-backward” condition. Recently, hybrid nanostructures, which combine plasmonic and dielectric components, have been widely explored for solar cell applications. In particular, spherical metallo-dielectric core-shell NPs have been shown to be interesting as scattering units for that purpose [28]. More complex metallo-dielectric core-shell geometries (specifically, conforming to a pyramidal shape) were introduced to improve light absorption in a broadband spectral region [29]. High efficiency was obtained in semitransparent organic solar cells by means of hybrid metal–nanoparticle–dielectric nanostructures [30]. Hybrid back reflectors have also been designed and fabricated to enhance the efficiency of thin film amorphous silicon solar cells [31]. Dielectric nanowire arrays made of HRID materials were proposed to improve the performance of solar cells due to their extraordinary light-trapping characteristics and high mobility for carriers. In addition, the combination of metallic NPs with HRID nanowires offers another way to enhance light scattering [32].

To analyze in more detail the utility of HRID NPs for solar cells applications, it is necessary to determine the fraction of radiation scattered into the photosensitive substrate when the particles are located on its surface. References [33,34,35,36,37,38] show the influence that dielectric and metallic substrates have on the optical response of plasmonic and HRID NPs. In particular, when HRID nanostructures are located on an HRI substrate, the incident radiation is preferentially scattered in the forward direction due to the coupling of Mie modes to the substrate [39]. As shown in reference [10], HRI substrates possess a high mode density, facilitating the coupling and modifying the width and spectral overlap of the electric and magnetic dipolar modes. This suggests that the scattering directionality conditions needs to be revisited for this situation [40].

In this work, we analyzed the electromagnetic response of different HRID nanoparticle geometries (isolated and aggregates) on monolayer and multilayer substrates to determine the most promising structure to enhance the efficiency of the radiation scattered into the substrate. Specifically, the number of layers composing the substrate [41,42,43,44,45,46,47,48,49,50,51,52,53], their thicknesses, and refractive index were optimized. We also show that HRID dimers on graded-index multilayer substrates can improve the efficiency of radiation scattered into the substrate when compared to that of isolated nanoparticles. These findings may be of great importance in the development of new technologies based on photovoltaic devices, such as solar cells. Focusing on that possibility, the study was carried out at the spectral region where the solar radiation is poorly absorbed by the silicon photosensitive layer (which most solar cells incorporate), i.e., the near-infrared (NIR) (*λ* = 800–1500 nm) range [22].

## 2. Results and Discussion

### 2.1. Scattering Geometry

**Figure 1:**
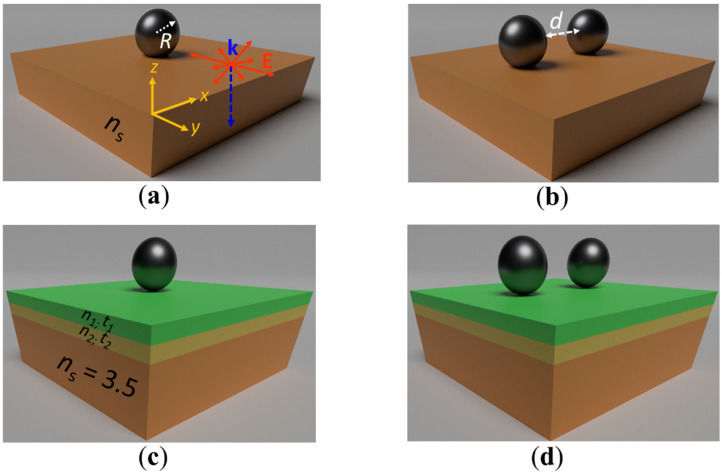
Scheme of the different analyzed HRID (silicon) configurations of spherical particles on a monolayer (**a**,**b**) and a multilayer (**c**,**d**) substrate of known optical properties. (**a**,**c**) Isolated particle, (**b**,**d**) dimer. All particles had a radius *R* = 150 nm. The gap distance between the particles was *d* = 10 nm. For the monolayer substrate, its refractive index was varied in the interval *n*_s_ ∈ [1.5–3.5]. The graded-index multilayer system consisted of two dielectric antireflection layers on a silicon substrate (*n*_s_ = 3.5). The thickness *t* and refractive index *n* of each layer were *t*_1_ = 185 nm, *t*_2_ = 104 nm and *n*_1_ = 1.396, *n*_2_ = 2.474, respectively. All geometries were illuminated by an unpolarized plane wave propagating along the -*z*-axis.

Different configurations of subwavelength HRID spherical nanoparticles located on a monolayer/multilayer dielectric substrate with known optical properties were analyzed. Figure 1 shows a scheme of the analyzed configurations: an isolated particle on a monolayer substrate (Figure 1a), a dimer on a monolayer substrate (Figure 1b), an isolated particle on a graded-index multilayer substrate (Figure 1c). and a dimer on a graded-index multilayer substrate (Figure 1d). All NPs had a radius of R = 150 nm. The HRID material considered was silicon and its optical properties, i.e., permittivity ε(ω), were taken from [54]. The dimer gap, denoted by d (see Figure 1b), was set to *d* = 10 nm in order to observe strong interaction effects between the nanoparticles, responsible for the “near zero-backward” condition [20,21]. The refractive index of the monolayer substrate was varied in the interval *n*_s_ ∈ [1.5–3.5]. The graded-index multilayer system consisted of two dielectric antireflective layers on a silicon substrate (without loss of generality, we considered its refractive index as *n*_s_ = 3.5 in the spectral range under study). The thickness *t* and refractive index *n* of each layer corresponded to *t*_1_ = 185 nm, *t*_2_ = 104 nm and *n*_1_ = 1.396, *n*_2_ = 2.474, respectively. Those parameters were chosen to minimize the reflection at the air–substrate interface (see Section 3).

All systems were excited by an unpolarized plane wave propagating along the negative direction of the *z*-axis to mimic sunlight illumination.

### 2.2. Radiation Scattered into a Monolayer Substrate

**Figure 2:**
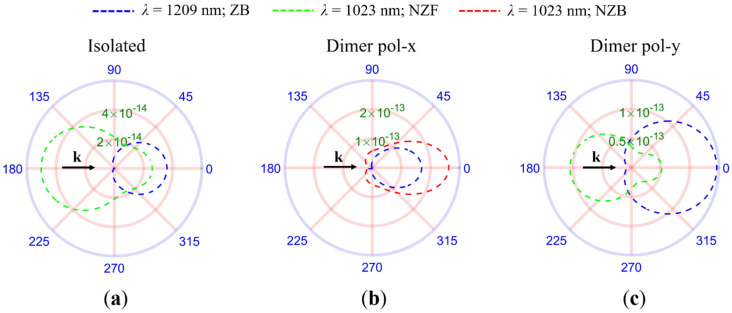
Scattering intensity diagrams in the scattering plane at the wavelengths where the scattering directionality conditions (SDCs) are observed for an isolated particle (**a**) and an HRID dimer (**b**,**c**). The NPs were illuminated by a linearly polarized plane wave propagating in the negative direction of the *z*-axis. (**b**) Polarization along the *x*-axis. (**c**) Polarization along the *y*-axis. ZB stands for zero-backward condition. NZF stands for near zero-forward condition and NZB stands for “near zero-backward” condition. The black arrow represents the direction of the incident radiation (k).

As we recently reported [20], aggregates of HRID NPs are more efficient at scattering the incident radiation towards the forward direction than isolated HRID NPs. This behavior comes from fulfilling the new scattering directionality condition, known as the “near zero-backward” condition, which is only observed when the particles interact strongly. This means that to observe this new directionality condition, the distance between the particles must be small and the polarization of the impinging radiation must be parallel to the main dimer axis. Also, the “near zero-backward” condition cannot be observed for isolated particles. In Figure 2a we show the scattering intensity patterns for an isolated silicon particle of radius *R* = 150 nm at the zero-backward and near zero-forward wavelengths. As mentioned in the introduction, with the prospect of using HRID NPs to improve the solar cells performance in mind, the particle radius was chosen to satisfy the SDCs in the near infrared (NIR) range. In Figure 2b,c we present the scattering intensity patterns for a silicon dimer of NPs for linearly polarized radiation parallel and perpendicular to the main dimer axis, respectively. The particle radius is also *R* = 150 nm and the gap distance *d* = 10 nm. The analyzed wavelengths are the same as the isolated particle case. It can be observed that, for both geometries (isolated particle and dimer), the zero-backward condition is fulfilled. However, at the near zero-forward condition wavelength for the isolated particle, the “near zero-backward” condition is attained for the dimer when illuminated by a linearly polarized plane wave along its main axis. This implies that, by using dimers with strong interaction effects between their components, it is possible to increase the fraction of radiation that is scattered towards the forward direction compared to that of isolated nanoparticles. However, the scattering directionality conditions are affected by the presence of the substrate, thus requiring the analysis of the influence of the substrate on the excitation of the new scattering directionality condition, and how this modifies the radiation that reaches the substrate. The incident radiation was assumed to be unpolarized, as if illuminated by sunlight [55]. This polarization contains all components of the electric field. This suggests that the increase in the fraction of radiation scattered into the substrate (*f*_subs_, see Section 3) is not as high as that for the case with the *x*-axis linearly polarized light, parallel to the dimer axis, but can still boost *f*_subs_ with respect to the isolated NP.

With the goal of determining the influence of the substrate on the “near zero-backward” condition, we studied the electromagnetic response of single and dimer HRID NPs configurations on the surface of a monolayer substrate, whose refractive index was varied in the range *n_s_* ∈ [1.5–3.5]. It was previously reported that for low values of the substrate refractive index (*n*_s_ *≈* 1.5), the SDCs can still be observed. However, as the refractive index of the substrate increases, the relative phase difference between the electric field originated by the electric and magnetic dipoles is less well defined, and the overlap in the far-field radiation patterns is reduced [10].

**Figure 3:**
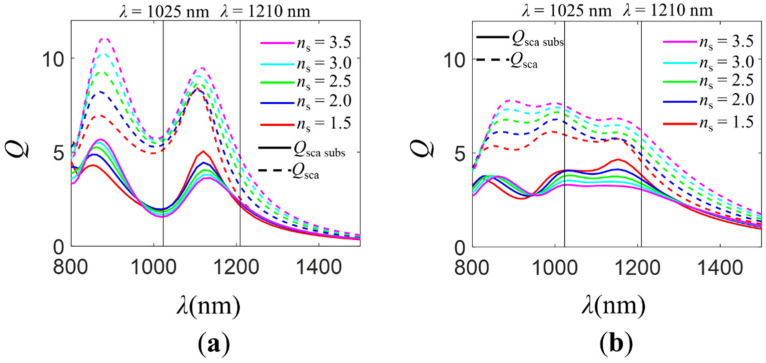
Total normalized scattering cross-section, *Q*_sca_ (dashed line), and normalized scattering cross-section for radiation scattered into the substrate, *Q*_sca subs_ (solid line), for different configurations of silicon NPs. (**a**) Isolated silicon NP of radius *R* = 150 nm. (**b**) Dimer of silicon NPs of radius *R* = 150 nm and gap distance *d* = 10 nm. The NPs were located on the surface of a monolayer substrate of known optical properties. The refractive index of the substrate was varied, *n*_s_ ∈ [1.5–3.5]. The structures were illuminated by an unpolarized plane wave propagating normally to the surface of the substrate. Vertical black lines correspond to the wavelengths where the zero-backward (*λ* = 1210 nm) and near zero-forward/“near zero-backward” (*λ* = 1025 nm) conditions were attained.

In Figure 3, we show the total normalized scattering cross-section, *Q*_sca_, and the normalized scattering cross-section for radiation scattered into the substrate, *Q*_sca subs_, (see Section 3) for the isolated NP (*R* = 150 nm, silicon) and the dimer (*R* = 150 nm, gap distance *d* = 10 nm, silicon) on different refractive index substrates (*n*_s_ ∈ [1.5–3.5]). For low refractive indices of the substrate (*n*_s_ *≈* 1.5), at wavelengths where the zero-backward condition is attained (𝜆 ≈ 1210 nm), most of the incident radiation is scattered into the substrate independently of the analyzed configuration (isolated NP or dimer). Nevertheless, at *𝜆* ≈ 1025 nm, the electromagnetic behavior is different depending on the geometry. For the isolated NP, the incident radiation is mostly scattered into the air. A minimum in the *Q*_sca subs_ spectrum is observed. The reason for this is the excitation at the near zero-forward condition wavelengths. For the dimer, at 𝜆 ≈ 1025 nm, *Q*_sca subs_ is higher than that of the isolated NP case. In fact, instead of a minimum, as for the isolated NP, a maximum is evidenced. In that spectral region, the “near zero-backward” condition is attained. As the refractive index of the dielectric layer takes higher values, the total normalized scattering cross-section increases in the analyzed spectral range *λ* ∈ [800–1500] nm. However, the normalized scattering cross-section for radiation scattered into the substrate decreases for both configurations at the wavelengths where the near zero-forward/“near zero-backward” condition is observed. This decrease is more significant in the case of the dimer. The fact that the portion of radiation reaching the substrate at the near zero-forward condition decreases for the isolated particle is mostly due to the increase of the reflection at the air–substrate interface. For the dimer, apart from the higher reflection, the difficulty in attaining the “near zero-backward” condition for high values of the substrate refractive index is another reason for the decrease in the amount of radiation scattered into the substrate. Consequently, the decrease is larger for the dimer than it is for the isolated NP. At the zero-backward condition spectral region, the scattered radiation into the substrate remains almost the same as that for the isolated particle, while it decreases for the dimer. Those results suggest that the SDCs depend more on the substrate presence in the case of the dimer than they do in the isolated NP. It is more difficult to achieve the required relative phase difference between the field created by the electric and magnetic dipoles when there are interaction effects between the particles responsible for the existing “extra” phase differences. This implies that for high refractive index substrates (such as those of the photovoltaic layer in solar cells), it is not possible to take advantage of the new scattering directionality condition “near zero-backward” for increasing the radiation scattered into the substrate. To exploit the “near zero-backward” condition to improve the performance of solar cells, in the next section, we analyze the electromagnetic behavior of isolated NPs and dimers on a graded-index multilayer substrate.

Table 1 shows the data corresponding to the fraction of radiation scattered into the substrate, *f*_subs_ (see Section 3), integrated over the spectral range *λ* ∈ [800–1500] nm, for different values of the substrate refractive index, *n*_s_. It should be noted that although *f*_subs_ provides a value between 0 and 1 for each wavelength, we integrated that value over the analyzed spectral range. This allowed us to make a comparison of the fraction of radiation scattered into the substrate in the NIR region between distinct configurations of NPs. As expected from the above discussion, as the refractive index of the substrate increased, the fraction of radiation scattered into the substrate decreased for both studied geometries, being larger for the dimer case. This makes the difference in the fraction of radiation scattered into the substrate between the isolated particle and the dimer smaller for large values of the substrate refractive index (Si, *n*_s_ ≈ 3.5), even though *f*_subs_ is larger for the dimer regardless of the substrate refractive index. This behavior is caused by two different factors. On the one hand, for low substrate refractive indices, the SDCs can be achieved. Meeting the “near zero-backward” condition leads to a higher *f*_subs_ for the dimer than for the isolated particle. Nevertheless, for high values of the substrate refractive index, SDCs are not attained. On the other hand, as the refractive index of the substrate increases, the reflection at the air–substrate interface increases. To avoid these two problems, it is necessary to consider a smoother transition in the refractive index between air and the photovoltaic substrate by using a multilayer substrate (see Section 2.3). In order to evidence the influence of the “near zero-backward” condition on the increase of *f*_subs_ values, we analyzed the *Q*_sca_, *Q*_sca subs_ spectra and *f*_subs_ for a dimer illuminated by a plane wave linearly polarized parallel (*x*-axis) or perpendicular (*y*-axis) to the axis that joins the NPs. The results are shown in Figure S1 and Table S1. For polarization along the *x*-axis, *f*_subs_ takes larger values than for *y*-axis polarization. This is due to the excitation of the “near zero-backward” condition. In fact, we observed that *f*_subs_ for unpolarized radiation (Table 1) presents intermediate values between those obtained for *x*-axis and *y*-axis linear polarization.

The enhancement in *f*_subs_ of the dimer compared to that of the isolated NP can be increased by considering arrays of HRID dimers. In addition, it is possible to use varying sizes of the NPs to compose the dimer at different unit cells, exciting the “near zero-backward” condition at different wavelengths. *f*_subs_ is thus increased in a broadband spectral region, improving the performance of photovoltaic devices.

### 2.3. Radiation Scattered into a Multilayer Substrate

In the previous section, it was reported that for high values of the substrate refractive index, the fraction of radiation scattered into the substrate (*f*_subs_) decreases for isolated NPs and aggregates. This is due to the impossibility of achieving the SDCs and high reflection at the substrate interface. To address this issue, we used a graded-index antireflective coating on the photosensitive layer (considered to made of silicon). The purpose of this coating is two-fold. First, by means of this graded-index coating, the reflection of the incident radiation is minimized. Second, the SDCs can be recovered as the difference in refractive index between air and the first antireflection layer is lower than that between air and silicon.

**Figure 4:**
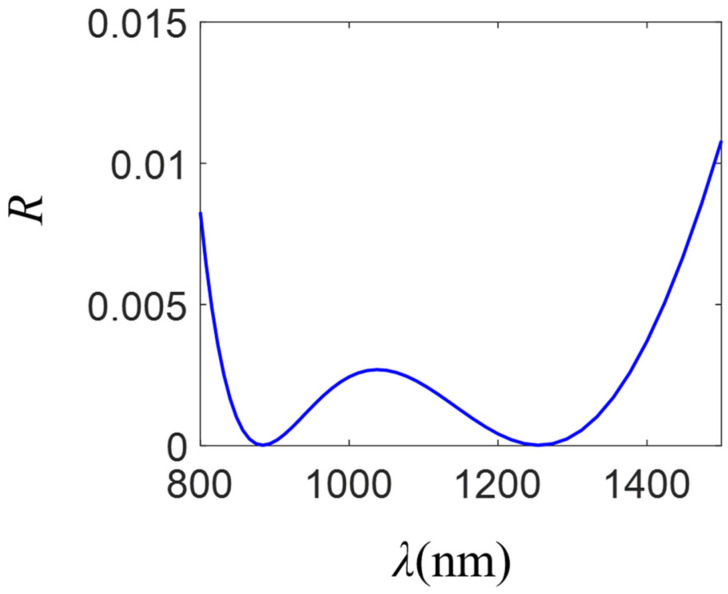
Reflection spectrum for a system comprising two antireflection dielectric layers located on a silicon substrate (*n*_s_ = 3.5). Their thicknesses (*t*) and refractive indices (*n*) correspond to *t*_1_ = 185 nm, *t*_2_ = 104 nm and *n*_1_ = 1.396, *n*_2_ = 2.474, respectively. The structure was illuminated by a plane wave propagating along the -*z*-axis.

The analyzed multilayer system consists of two antireflection dielectric layers on a silicon substrate. The structure that minimizes the reflection was obtained by following the procedure explained in the Section 3. In Figure 4 we show the reflection spectrum for a system comprising a two-layer antireflection coating on a silicon substrate. The thicknesses (*t*) and refractive indices (*n*) of the layers correspond to *t*_1_ = 185 nm, *t*_2_ = 104 nm and *n*_1_ = 1.396, *n*_2_ = 2.474, respectively.

**Figure 5:**
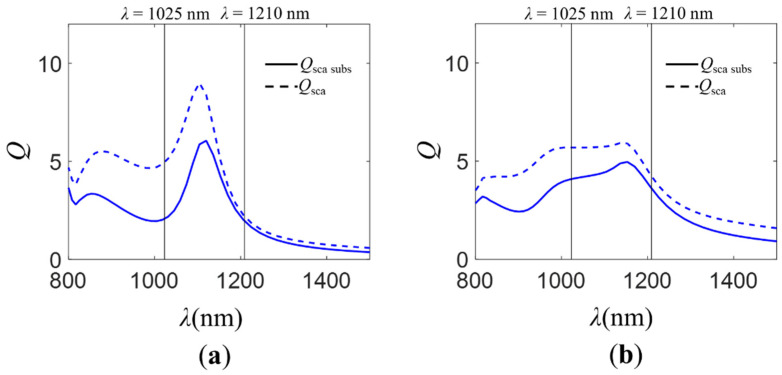
Total normalized scattering cross-section, *Q*_sca_ (dashed line), and normalized scattering cross-section for radiation scattered into the substrate, *Q*_sca subs_ (solid line), for different configurations of HRID particles on a graded-index multilayer substrate. (**a**) Isolated silicon NP of radius *R* = 150 nm. (**b**) Dimer of silicon NPs of radius *R* = 150 nm and gap distance *d* = 10 nm. The substrate consists of two dielectric layers of thicknesses (*t*) and refractive indices (*n*) *t*_1_ = 185 nm, *t*_2_ = 104 nm and *n*_1_ = 1.396 and *n*_2_ = 2.474, respectively, on a silicon layer (*n*_s_ = 3.5). The structures were illuminated by an unpolarized plane wave propagating normally to the surface of the substrate. Vertical black lines correspond to the wavelengths where the zero-backward (*λ* = 1210 nm) and near zero-forward/“near zero-backward” (*λ* = 1025 nm) conditions are attained.

In Figure 5 we show the total normalized scattering cross-sections, *Q*_sca_ and *Q*_sca subs_, for a single and a dimer of HRID NPs on the described multilayer substrate (see Section 3). When the multilayer substrate is considered, the *Q*_sca_ and *Q*_sca subs_ spectra are similar to those obtained for a monolayer substrate of low refractive index. This suggests that, thanks to the graded-index antireflective layers, it is possible to recover the SDCs and take advantage of the “near zero-backward” condition. In addition, the reflection at the air–substrate interface is minimized.

In Table 2 we show a comparison of the fraction of radiation scattered into the substrate *f*_subs_ for the different analyzed configurations, integrated over the spectral range *λ* ∈ [800–1500] nm, when they are located on different substrates. Specifically, we studied two monolayer substrates of refractive indices *n*_s_ = 1.5 (SiO_2_)and 3.5 (Si), respectively, and a multilayer substrate that comprises a two-layer antireflection coating on a photovoltaic silicon substrate. It was observed that by using the antireflection coating, the fraction of radiation scattered into a silicon substrate was similar to that of a SiO_2_ monolayer substrate, improving the results obtained for the case of a bare silicon substrate. The same conclusions were found for a dimer illuminated by a plane wave linearly polarized along the *x*- or *y*-axis (see Figure S2 and Table S2). Furthermore, the values of *f*_subs_ were higher for the dimer than for the isolated NP. This suggests that aggregates of HRID particles are more efficient than single NPs for redirecting the incident radiation into the photovoltaic substrate. Therefore, those configurations can be useful for improving the performance of current solar cells.

## 3. Materials and Methods

### 3.1. Fraction of Radiation Scattered into the Substrate

Numerical solutions were obtained by means of a FDTD solver (Lumerical 2016b) [56]. For the illumination configuration, two total field scattered field (TFSF) sources surrounding the particles were used. These sources launched a broadband plane wave from the top of the system under normal incidence. The two sources were 90° out of phase and orthogonally polarized with respect to the other. This allowed us to define circularly polarized light for the incident radiation. In our simulations, we assumed that particles were illuminated with circularly polarized light to emulate the more realistic unpolarized illumination. The most common way of simulating unpolarized light consists of performing two different simulations for orthogonal input electric fields and taking the mean of the results of such simulations. However, executing only one simulation with circularly polarized light is more efficient from a computational point of view.

By means of power monitors located surrounding the sources, the total normalized scattering cross-section *Q*_sca_ (defined as the total scattering cross-section *σ*_sca_ divided by the geometrical cross-section *σ*_geo_, *Q*_sca_ = *σ*_sca_/*σ*_geo_) [57] was obtained. The scattered power was calculated by integrating the Poynting vector of the scattered field over the monitors (i.e. a box surrounding the sources) [58]. By dividing the scattered power by the incident intensity, we obtained the scattering cross-section, *σ*_sca_. The power scattered into the air or the dielectric substrate can be calculated by integrating the scattered power over the relevant parts of the simulation box. It is worth mentioning that for the multilayer substrate, the power scattered into the substrate was obtained by integrating only over the photosensitive layer (i.e., the silicon layer) because only photons absorbed in this layer contribute to increasing the electric current. The fraction of radiation scattered into the substrate *f*_subs_, which has been demonstrated in previous works [10,58] to be the most important factor in determining the path length enhancement of a light-trapping structure, is defined as the power scattered into the substrate divided by the total scattered power *Q*_sca subs_/*Q*_sca_. Perfectly matched layers (PMLs) were employed for absorbing the scattered field. For the solutions to converge fully, a 2 nm mesh refinement for the region surrounding the particles was used.

To obtain the results shown in the Supplementary Information, the above-described procedure was followed. However, only one total field scattered field (TFSF) source surrounding the particles was considered, i.e. a plane wave linearly polarized along the *x*-axis or *y*-axis under normal incidence.

The scattering far-field diagrams were calculated through a finite element method, implemented in the commercial software COSMOL Multiphysics 5.0 [59]. Using the Radio Frequency Module in COMSOL, the Maxwell equations alongside the boundary conditions can be solved in the frequency domain. The electric field in the far-field was obtained using the Stratton–Chu formula [60]. To perform these simulations, the structure was illuminated with a plane wave propagating along the negative *z*-axis and linearly polarized along the *x*- or *y*-axis. The particles were surrounded by a sphere of air with a radius of *λ* (wavelength of the incident radiation). To ensure numerical convergence of the results, the tetrahedral mesh was chosen to be sufficiently fine. Thus, the mesh of the surrounding air medium was less than *λ*/4 and the mesh of the nanoparticles was lower than *λ*/10. The homogeneous spherical region of air was surrounded by a perfectly matched layer (PML) with a thickness of *λ*/4.

### 3.2. Optimization of the Multilayer Substrate

Graded-index multilayer substrates were analyzed. In particular, two dielectric layers were considered with the aim of minimizing the reflection of the incident radiation at the interface of a silicon substrate, which acts as the photosensitive layer. In order to determine the thickness (*t*) and refractive index (*n*) of each one of the layers, the reflection spectra corresponding to different *t* and *n* values were calculated using the complex-matrix form of the Fresnel equations (transfer matrix method) [61,62]. Both parameters were varied by means of an iterative process that consists of the following steps:

1. A loop of values for both parameters (*t* and *n*) for each of the layers was selected.
2. For each simulation (corresponding to a certain *t* and *n* value for each one of the layers), the area under the curve corresponding to the reflection spectrum was analytically calculated.
3. A bespoke MATLAB program returned the thickness and refractive index values that provided the minimum reflection (minimum value in step 2).

The number of layers was chosen to achieve minimum reflection while considering a realistic experimental configuration.

## 4. Conclusions

The electromagnetic behavior of different configurations of HRID spherical particles (isolated and dimers), on monolayer and graded-index multilayer substrates, was analyzed to find a novel configuration capable to increase the fraction of radiation scattered into a silicon substrate. In particular, a geometry that consisted of an HRID dimer on a graded-index multilayer substrate was proposed to boost the performance of photovoltaic devices, i.e., solar cells.

As demonstrated in previous works [20,21], dimers with strong interaction effects between their components are more efficient at redirecting the incident radiation in the forward direction than are single nanoparticles. This is due to the appearance of a new scattering directionality condition known as the “near zero-backward” condition.

We reported that the electromagnetic response of the different analyzed configurations is affected by the optical properties of the substrate. In particular, we showed that for low values of the substrate refractive index (*n_s_* ≈ 1.5), the SDCs can still be observed. At the wavelengths for which the zero-backward condition holds, the incident radiation is scattered towards the substrate, being this effect valid for both, the isolated nanoparticle and the dimer. At the wavelengths for which the near zero-forward condition is attained for isolated particles, the incident radiation is mostly back-scattered. At those wavelengths, for the dimer case, the “near zero-backward” condition is achieved, increasing the radiation scattered into the substrate. As a result, by working with aggregates, it is possible to improve the coupling of the incident radiation with the substrate. Nevertheless, as the refractive index of the substrate increases, reaching values similar to those of silicon (the most widely used material in the photovoltaic layer of solar cells), the SDCs are no longer attainable. In addition, the reflection at the substrate interface is increased. Consequently, aggregates are not efficient scattering units to redirect the incident radiation towards the substrate. To address this issue, we proposed employing a graded-index antireflection coating on the silicon photovoltaic layer. The multilayer substrate allowed for the recovery of the SDCs and the diminishment in the reflection of the incident radiation at the substrate interface. For a dimer on a system comprising two antireflection layers on a silicon substrate, *f*_subs_ takes similar values to those obtained for the dimer on a monolayer substrate of low refractive index. This makes the analyzed aggregates configurations on a multilayer substrate efficient building blocks for improving the efficiency of solar cells. Furthermore, *f*_subs_ can be increased by considering arrays of HRID dimers. Then, by designing metasurfaces, in which different unit cell have dimers of varying sizes, the “near zero-backward” condition can be satisfied at different wavelengths, boosting *f*_subs_ in a broadband spectral range.

## Figures and Tables

**Table 1:** Fraction of radiation that is scattered into the substrate, *f*_subs_, integrated over the analyzed spectral range (*λ* ∈ [800–1500] nm) for different substrate refractive indices, *n*_s_.

Geometry	*n*_s_ = 1.5	*n*_s_ = 2.0	*n*_s_ = 2.5	*n*_s_ = 3.0	*n*_s_ = 3.5
Isolated NP	32.2	30.3	27.8	25.7	24.0
Dimer	34.7	32.4	29.5	27.1	25.1

**Table 2:** Fraction of radiation scattered into the substrate, *f*_subs_, integrated over the spectral range *λ* ∈ [800–1500] nm for the different analyzed particle configurations. We show the cases corresponding to two monolayer substrates made of SiO_2_ (*n*_s_ = 1.5) and silicon (*n*_s_ = 3.5) and a multilayer substrate constituted by two antireflection layers (A.L.) on a silicon substrate.

Geometry	*n*_s_ = 1.5	*n*_s_ = 3.5	Two A.L. *n*_s_ = 3.5
Isolated NP	32.2	24.0	31.4
Dimer	34.7	25.1	34.0

## Data Availability

Data are available upon reasonable request.

## Supplementary Materials

The following are available online. Figure S1: Cross-sections for an HRID dimer on monolayer substrates as a function of the polarization of the incident radiation, Table S1: Fraction of radiation scattered into monolayer substrates by an HRID dimer as a function of the polarization of the incident radiation, Figure S2: Cross-sections for an HRID dimer on a multilayer substrate as a function of the polarization of the incident radiation, Table S2: Fraction of radiation scattered into a multilayer substrate by an HRID dimer as a function of the polarization of the incident radiation.
