# Supplementary material for: Broadband Unidirectional Forward Scattering with High Refractive Index Nanostructures: Application in Solar Cells
Source: Molecules. 2021 Jul 22;26(15):4421. doi: 10.3390/molecules26154421 (PMC8347201; doi:10.3390/molecules26154421)
Supplement: Supplementary file 1 [file molecules-26-04421-s001.zip › molecules-1276088-supplementary.pdf]

molecules

Article

Broadband Unidirectional Forward Scattering with High Refractive Index Nanostructures: Application in Solar Cells

Ángela Barreda [1,*], Pablo Albella [2], Fernando Moreno [2] and Francisco González [2]

[1] Institute of Applied Physics, Abbe Center of Photonics, Friedrich Schiller University Jena, Albert-Einstein-Str. 15, 07745 Jena, Germany
[2] Group of Optics, Department of Applied Physics, University of Cantabria, Cantabria 39005, Spain
* Correspondence: angela.barreda@uni-jena.de

Citation: Barreda, Á.; Albella, P.; Moreno, F.; González, F. Broadband Unidirectional Forward Scattering with High Refractive Index Nanostructures: Application in Solar Cells. *Molecules* **2021**, *26*, 4421. https://doi.org/10.3390/molecules26154421

Academic Editor: Minas M. Stylianakis

Received: 10 June 2021
Accepted: 20 July 2021
Published: 22 July 2021

Publisher's Note: MDPI stays neutral with regard to jurisdictional claims in published maps and institutional affiliations.

Copyright: © 2021 by the authors. Licensee MDPI, Basel, Switzerland. This article is an open access article distributed under the terms and conditions of the Creative Commons Attribution (CC BY) license (http://creativecommons.org/licenses/by/4.0/).

1. Radiation Scattered into a Monolayer Substrate

The electromagnetic behavior of dimers of High Refractive Index Dielectric (HRID) nanoparticles (NPs) on monolayers and multilayers substrates was analyzed for different illuminations of the incident radiation. In the manuscript, we showed the results for unpolarized radiation. To compare, in Supplementary Figure 1, we present the total normalized scattering cross-section Q_{sca} and the normalized scattering cross-section for radiation scattered into the substrate $Q_{sca\ subs}$ for a dimer illuminated with a plane wave linearly polarized parallel to the axis that joins both components of the dimer, x-axis, (see Figure 1 for axis orientation) or perpendiclar to it, y-axis. This polarization analysis was not performed for the isolated NP. Due to symmetry reasons, the same results of Q_{sca} and $Q_{sca\ subs}$ are obtained for linearly polarized (along x- or y-axis) and unpolarized radiation.

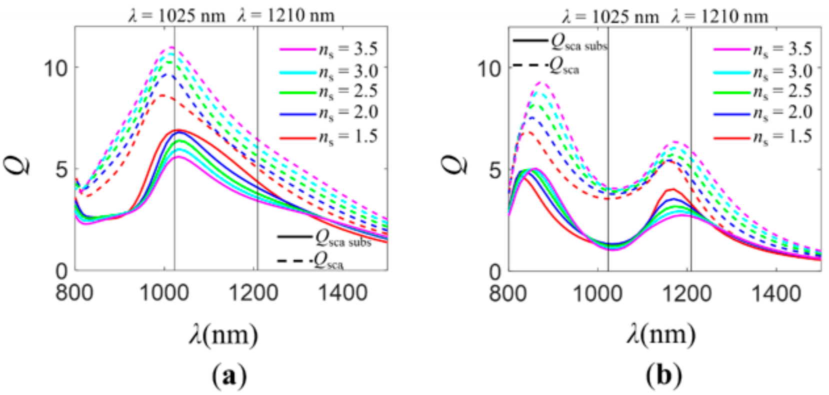

Figure S1. Total normalized scattering cross-section Q_{sca} (dashed line) and normalized scattering cross-section for radiation scattered into the substrate $Q_{sca\ subs}$ (solid line) for a dimer of HRID NPs, when different polarizations of the exciting radiation are considered. (**a**) Plane wave linearly polarized parallel to the x-axis (axis joining both components of the dimer). (**b**) Plane wave linearly polarized along the y-axis. The propagation of the plane wave was normal to the substrate. The particle radius and gap distance are R = 150 nm and d = 10 nm, respectively. The NPs were located on the surface of a monolayer substrate of known optical properties. The refractive index of the substrate was varied $n_s \in$ [1.5-3.5]. Vertical black lines correspond to the wavelengths where the Zero-Backward (λ = 1210 nm) and near Zero-Forward/"near Zero-Backward" (λ = 1025 nm) conditions are attained.

In Supplementary Table 1, we show the data corresponding to the fraction of radiation scattered into the substrate f_{subs} integrated over the spectral range $\lambda \in$ [800-1500] nm, for different values of the substrate refractive index n_s. We performed those computations for the configurations represented in Supplementary Figure 1 (incident radiation linearly polarized along the *x*- and *y*-axis). Like for the case of unpolarized electromagnetic

radiation, as the refractive index of the substrate increases, the fraction of radiation scattered into the substrate decreases for both analyzed polarizations. This decrease is larger for the *x*-axis polarization. The reasons for this decrease are the same as those introduced in the main manuscript. In addition, it is possible to observe that f_{subs} for the unpolarized radiation (Table 1) takes intermediate values between those obtained for *x*-axis and *y*-axis linear polarization. By means of the comparison of both polarizations, it is reported that the fraction of scattered radiation into the substrate is larger for the *x*-axis polarization than for the *y*-axis polarization. This is due to the excitation of the "near Zero-Backward" condition, which is only attained for strong interactions effects between the NPs, i.e., for *x*-axis polarization.

Table S1. Fraction of radiation that is scattered into the substrate, f_{subs}, integrated over the analyzed spectral range ($\lambda \in$ [800-1500] nm) for different substrate refractive indices n_s.

Dimer	n_s=1.5	n_s=2.0	n_s=2.5	n_s=3.0	n_s=3.5
x-axis pol.	36.5	33.5	30.3	27.6	25.4
y-axis pol.	31.4	29.8	27.4	25.3	23.5

2. Radiation Scattered into a Multilayer Substrate

In Supplementary Figure 2, we show the total normalized scattering cross-section (Q_{sca}) and the normalized scattering cross-section for radiation scattered into the substrate ($Q_{sca\ subs}$) for a dimer of HRID spherical particles on a silicon substrate (n_s = 3.5), over which two antireflection layers are located. The dimer was illuminated with a plane wave propagating normal to the substrate and linearly polarized along the *x*-axis (parallel to the axis joining both components of the dimer) or along the *y*-axis. For this graded-index multilayer substrate, the Q_{sca} and $Q_{sca\ subs}$ spectra are similar to those obtained for a monolayer substrate of low refractive index ($n_s \approx 1.5$).

In Supplementary Table 2, we present a comparison of the fraction of radiation scattered into the substrate f_{subs} by a dimer of spherical NPs, integrated over the spectral range $\lambda \in$ [800-1500] nm, when the NPs are located on distinct substrates. Specifically, we studied two monolayer substrates of refractive indices n_s = 1.5 and 3.5, and a multilayer substrate that comprises a two-layer antireflection coating on a photovoltaic silicon substrate (n_s = 3.5). The analysis was carried out for different polarizations of the incident radiation (plane wave linearly polarized along the *x*- and *y*-axis). Through the obtained results, it is observed that, by using the antireflection coating, the fraction of radiation scattered into a silicon substrate is similar to that attain for a SiO_2 ($n_s \approx 1.5$) monolayer substrate, improving the results with respect to the case of a bare silicon substrate. The values of f_{subs} are higher for the *x*-axis polarization than for *y*-axis polarization, due to the excitation of the "near Zero-Backward" condition.

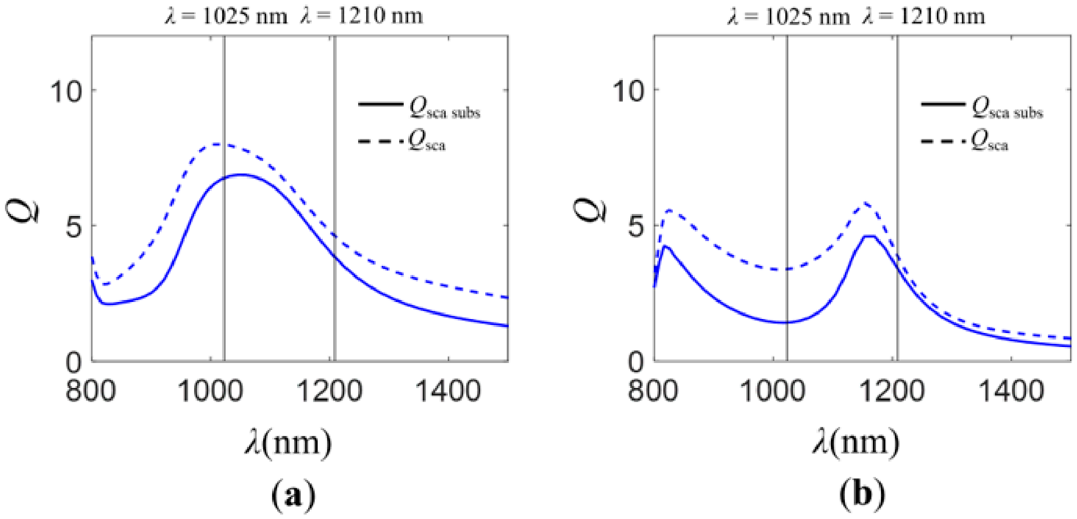

Figure S2. Total normalized scattering cross-section Q_{sca} (dashed line) and normalized scattering cross-section for radiation scattered into the substrate $Q_{sca\ subs}$ (solid line) for a dimer of HRID particles on a graded-index multilayer substrate when different polarizations of the incident radiation are considered. (**a**) Plane wave linearly polarized parallel to She *x*-axis (axis joining both components of the dimer). (**b**) Plane wave linearly polarized along the *y*-axis. The propagation of the plane wave was normal to the substrate. The particle radius and gap distance are R = 150 nm and d = 10 nm, respectively. The substrate is constituted by two dielectric layers of thicknesses (t) and refractive indices (n) t_1 = 185 nm, t_2 = 104 nm; n_1 = 1.396 and n_2 = 2.474 on a silicon layer (n_s = 3.5). Vertical black lines correspond to the wavelengths where the Zero-Backward (λ = 1210 nm) and near Zero-Forward/"near Zero-Backward" (λ = 1025 nm) conditions are attained.

Table S2. Fraction of radiation scattered into the substrate f_{subs} integrated over the spectral range $\lambda \in$ [800-1500] nm. We show the cases corresponding to two monolayer substrates made of SiO_2 (n_s = 1.5) and silicon (n_s = 3.5) and a multilayer substrate constituted by two antireflection layers (A. L.) on a silicon substrate.

**Dimer**	n_s**=1.5**	n_s**=3.5**	**Two A. L.** n_s**=3.5**
x-axis pol.	36.5	25.4	35.3
y-axis pol.	31.4	23.5	31.7
